# Barriers of child adoption in infertile couples: Iranian’s views 

**Published:** 2012-09

**Authors:** Mahshid Bokaie, Tahmineh Farajkhoda, Behnaz Enjezab, Pooran Heidari, Mojgan Karimi Zarchi

**Affiliations:** 1 *Department of Nursing and Midwifery, Faculty of Nursing and Midwifery, Shahid Sadoughi University of Medical Sciences, Yazd, Iran.*; 2 *Department of Obstetrics and Gynecology, Faculty of Medicine, Shahid Sadoughi University of Medical Sciences, Yazd, Iran.*

**Keywords:** *Child Adoption*, *Infertility*, *Barriers*, *Iranian views*, *Cultural and social barrier*

## Abstract

**Background:** There are many reasons why some couples do not become parents. Some are infertile, some do not want kids, children can be in a social context unacceptable and for others different life goals are more important.

**Objective: **This study was designed to determine barriers of child adoption in infertile couples in Iran.

**Materials and Methods:** This cross-sectional study was carried out at Iran from April 2010 to June 2011. The research program was comprised consecutively in 240 infertile couples. Experts in Guidance and Counseling vetted the instrument and set that it has content validity. Test re-test reliability was conducted by the investigators using a sample of 20 couples who have filled questionnaire.

**Results:** Although 230 (96%) of the respondents heard of child adoption, only 89 (37.3%) of couples knew correct meaning of child adoption. Fifty four women (24%) knew how to adopt a baby while the rest did not; 196 (82%) respondents expressed their unwillingness to adopt a baby. Hoping of childbearing (78%) was the main barrier to adopt a child.

**Conclusion:** The barriers mentioned were cultural practices, stigmatization, financial implications, and technical problems. Most of the infertile Iranian couples prefer to stay even so without children or think about new treatment.

## Introduction

Today more than 70 million couples suffer from infertility around the world. Nearby 10-15 percent of couples have a problem to become pregnant (1). near 25% of the Iranian couples experience infertility in their life and 3.4% experience this problem at any time (2). Bilateral tubal obstruction is the most common cause of infertility (3). In different cultures across the world, many couples without or wishing to have more children emprise to adopt child or foster orphans (4, 5). 

Child adoption is seen as an act of responsibility of children who are not biologically related to a couple (6). The infertile couples suffer from the conflux of individual, interpersonal, social, and spiritual expectations thus bringing a sense of breakdown to them. In some cultural settings in Africa, childless couples are even not permitted to take leading role in important family functions and procedures. 

In addition, these couples are often socially ostracized by their immediate families. These challenges are not only limited to the developing world (7). Couples in lower socio-economic groups are less likely to consult their doctors when they experience difficulties in fertility (6, 8). 

With the quick increase in international adoption, people in Western Europe and North America adopt children from countries in the south and the former Soviet domain(9). The main predictor of patient centered care and professional psychosocial services for couples was high infertility related tension in the marital, personal and social amplitude(10). 

Inability to fertility is frequently considered as a personal lugubrious and a curse for the couple, impression on the all family and even the local society. Negative psychosocial subsequences of infertility are common and often severe (11-13). Infertility is one of reproductive health problem in developing countries, which frequently carries negative psychosocial complications. 

In many cases, infertility is caused by sexual transmitted diseases (14). Infertility remains a large-scale health challenge with upsetting psychosocial penalty in many Iranian communities. Child adoption that may give out as a different strategy for the exaggerated couples is not widely accomplished (7). Sterility is a potential cause of marital unsteadiness especially in the African culture where it is used as a parameter for marital triumph. This is because a wedding without children is likely to be nerve-racking for couples due to pressures from relatives especially cheering the husband to take up a second wife where the woman is supposed to be barren (6). 

In our culture child adoption is a new option; person who wants to adopt a child may be from child’s family or outside of the relatives (15)*.* Islam has a large number of rights about child adoption. In both Islamic religious law and the global statement, the child rights are very important (16). According to Behzisti organization (2011), the legal organ for child adoption in Iran, the number of children for adoption is much less than requests. More investigation needs to show child adoption challenges and researches improve and facilitate existing condition (4). Complexity of legal process for child adoption in Iran is considered as another barrier. 

According to special Iranian socio-cultural background, there is not enough study towards barriers of child adoption in Iran. Therefore, this study was designed to determine barriers of child adoption in infertile couples in Iran.

## Materials and methods

This cross-sectional study was carried out in Shahid Sadougi University of Medical Sciences in Yazd, Iran from April 2010 to June 2011. In this study, 240 infertile couples attending the Infertility Center of Yazd were participated in the study, the ethics committee of Shahid Sadoughi of Medical Sciences was approved the study project. Informed consent was obtained from all participants.

Conventional sampling was selected for data collection. Data was collected by structured questionnaire. The questionnaire was prepared using related scientific investigations, pilot study and viewpoints of experts in this area. Pilot study was conducted through recruitment of 20 couples in order to revise the questionnaire. Face and content validity of the questionnaire was confirmed by eight faculty members of the university. 

The first part of the questionnaire was regarding to demographic data (age, sex, educational and employment status). Second section consisted of several questions towards infertility status (infertility duration, type of infertility and infertility causes). The third section was related to awareness of child adoption and the last part of questionnaire was focused on the barriers of child adoption. Two health care workers were trained in order to complete the questionnaires. Inclusion criteria were Iranian ethnicity, length of infertility ≥5 years after marriage and infertility approval by physician.

**Statistical analysis**

The data were analyzed by means of simple percentages for categorical variables, descriptive statistics for continuous variable, and using t-tests and chi-square tests for qualitative variables at the 95% confidence level.

## Results

Two hundred forty questionnaires were analyzed. The mean age of women was 27.5±4.8 and their spouses’ age was 32.7±5.6 years. There was no significant difference between couples age and tendency to child adoption. Most of them (38.8% of female and 40% of male) had diploma degree (Table I). There was no significant difference between couples educational status and leaning to child adoption. 

Types of infertility were another variable of the study. Although in five cases with male factor infertility, more interest to child adoption was observed, there was no significant difference between types of infertility and any desire to child adoption (Table II). In most of them (52.9%), duration of infertility was 1-5 years (Table III). it was very interesting to us because between duration of infertility and willing to child adoption there was no significant difference.

Participants’ knowledge towards child adoption was assessed in this study. Although 230 (96%) of the participants heard about child adoption, most of the couples (151 couples, 62.7%) did not know the correct meaning of child adoption. Only fifty-four (24%) couples knew child adoption process. One hundred ninety six (82%) participants declared their unwillingness to adopt a baby. They thought fatalism was something that God wants (Table IV). 

Question about child adoption were not gracious for them in 172 cases (72%). They were shy to tell about their problem to their friends and others. If they adopted child, they would prefer infancy age (n=85, 35.6%) and in 105 cases (43.6%) his or her age would not be important for them. In 173 cases (72.3%) sex of baby was not important for them. 

To the question whether they loved the child as the real parents if they adopted a child, one hundred and seven cases (44.6%) said yes, 38 (15.8%) said no and 162 (37.6%) were uncertain about their response. In 109 cases, they did not know how to adopt a baby. In 83 cases (34.7%) legal process of adoption was not acceptable for them. 

Most of them believed babies bring happiness to their home. The main reasons mentioned by the participants were unwilling to adopt a child: We are hoping of childbearing (78%), adoption is not appropriate way for solving our infertility problem (65%), adoption is not acceptable psychologically for us (52%), and fear of unknown parental background of child (32%) (Table V).

**Table I:** Educational status

	**Male N (%)**	**Female N (%)**
Under Diploma	70 (28.8)	84 (35)
Diploma	96 (40)	93 (38.8)
Academic	64 (27)	53 (22)
No answer	10 (4.2)	10 (4.2)
Total	240 (100)	240 (100)

**Table II:** Cause of infertility

	**NO**	**%**
Female factor	90	37.6
Male factor	87	36.5
Both female and male factor	11	4.7
Unknown problem	50	21.2
Total	238	100

**Table III:** Duration of infertility

	**NO**	**%**
1-5 year	126	52.9
6-10 year	56	23.5
Above 11 year	48	20
No answer	8	3.5
Total	238	100

**Table IV:** General knowledge about child adoption

**Questions**	**Answer**	**No**	**%**
Did you ever heard about adoption?	Yes	230	96
Do you know the meaning of child adoption?	Correct	89	37
You know the legal child adoption process in Iran?	Correct	54	24
Are you willing to adopt a child?	Yes	37	15

**Table V:** Child adoption barrier in infertile couples

**Reason**	**No**	**%**
We are hoping of childbearing	187	78
Adoption will not solve our problem	156	65
Child adoption is psychologically unacceptable to us	124	52
Unknown parental history and pedigree	115	48
Adoption is not acceptable in our culture	98	41
Fear of future	76	32
Adopted child is not similar to us	74	31

## Discussion

The present study’s results regarding child adoption in infertile women were educational status, cause of infertility, duration of infertility and general knowledge about child adoption. In African and many Asian countries there are many children for adoption consequent upon low contraceptive methods (17). However, in Iran, the number of children for adoption is much less than requests and in contrast, in Western countries child adoption is a very known practice and many agencies are ready to assist the willing couples (18). 

Child adoption should be considered as an important approach for negative consequences of infertility (4). The aim of this study was to assess the barriers of child adoption according to Iranians’ views. Tazeen *et al* study (2007) showed adoption was the last option for infertile couples in India that half of the female who had either thought of or had adopted a kid were uneducated. 

The high level of illiteracy affected not only their health looking for performance but their condition in the family too. Therefore, they were not in a position to make a decision. In our results, 35% of females and 29% of males had primary level of education (19). According to our study, the level of education did not influence the child adoption. Aghanwa believed infertile women in Nigeria living through poor marital kinship and low socioeconomic level were not ready for child adoption. It may be influenced from educational level but in our study this parameter did not affect the child adoption (20).

According to the study results, there was no relationship between types of infertility and desire to child adoption. Even in the five cases of male barrenness, women usually stand the negative consequences of their failure to conceive. In Ombele *et al* (2008) male factor infertility created a negative sensation and caused a guilty response in women because they thought they are unable to concept (14). Preventive and health-giving services for infertility have not yet been a right of way in India despite the substance of motherhood. The problem of infertility in India has to be interpreted in a context of deficiency, class and gender disparity and unequal access to health-care income (19).

Although the great part of infertile Nigerian women heard of child adoption, only an underground knew its factual meaning. Just about one-third of them were interested in adoption as a treatment option for their infertility (21). Although 96% of our participants heard of child adoption, majority of them (62.7%) were not familiar with its correct meaning. The common barriers of Nigerian women were child adoption will not solve our problem while it is the second barrier of Iranian women. In our study, 85% did not agree with child adoption and, in Nigeria 59.3% did not emprise to child adoption. 

Ozugwu’s finding was similar to our investigation. Lack of knowledge about child adoption was seen in African population. The respondents with little information might be those whose doctors hope to cure of their infertility and they will become pregnant (21). Our finding show that level of knowledge about child adoption is low in Iran. 

Family with adopted children might be feeling many troubles, but kids bring happiness to their home and when one of the parties is not very responsible, they may also be a reason of argument between partner and wife particularly (6). Our results showed that most couples believed babies bring happiness to their home. This confirms Dimkpa (2010) findings. The main mentioned barrier in this study was hoping to be pregnant by new medication of infertility. 

According to Oladokun (2010) cultural practices, stigmatization, financial implications, and technical problems were the barriers of child adoption in Nigeria. In Western countries language and cultural barrier has been proposed. Measures recommended to curb these negative attitudes were advocacy, community movement and performance of supportive law that will protect all parties involved (7). In our country's cultural and social barriers are also the other major causes of child adoption and Oladokun results confirms this part of our finding.

In attempt to discover a solution to their difficulty regarding child adoption in Iran, most couples are shy in seeking help. It is important for such couples to endure counseling which will help them in choosing from different alternatives open to them. Ali *et al* (2007) shows adoption is the last option for infertile couples in India (19). Negative family attitude toward child adoption and shy from their problem were reported in India (22).

In 173 cases (72.3%), sex of baby was not important for them in current study, but in Ezugwu *et al* study, 59% prefer male baby. In our study, 44.6% loved the child as a real parent but in Ezugwu study, all of them loved the child as a real parent. Near 50% of cases, they did not know how to adopt a baby. In Ezugwu study, 78% did not know how to adopt a baby. In 83 (34.7%) cases legal process of adoption were not acceptable for them. In Ezugwu study 99% of them are willing to go through the legal process involved in adoption (21). 

Willing to self-motherhood was determined as one of the most important barrier in this study that addressed by more than half of participants. According to Bharadwaj study (2003), child adoption is not acceptable for many families in Eastern countries. Motherhood is the most important purpose for traditional female in this country. Children, particularly boys, give the woman status as well as psychological and exciting security within the patriarchal family unit. Infertility is related with social isolation and sometimes violent behavior (22, 23). 

These results were compared with our finding regarding participant’s traditional attitude toward self-motherhood as dominant barrier in Iranian family and other Eastern countries. In the UK, supporting initiatives have encouraged the bigger use of adoption as a key to the care of children who cannot live with their origin families. This force for 'permanence' has been welcomed by several, but has also given rise to argue (24). 

It comes at a time of essential change in child adoption practice as, adaptive, Irish couples look outside the court case for how become their family. In Karpel research, 49% of these couples are not in favor of adoption, especially when they have already had children through medical help (15). All these results showed child adoption in Asia and Latin America has very different motivation versus Western country.

Despite of rapidly reducing demand for biological kids in most Western countries, people motivates to adopt children who are not geographically linked to them (18). Biological kids are very important for Iranian, Nigerian, Indian and many Eastern families. 

## Conclusion

Most of the infertile Iranian couples will prefer to stay even so without children or to think about new treatment. When the history of infertility was so prolong they probably have to hear from their physician that the treatments are definitively impossible to become completely involved in the adoption process. 

Hoping of childbearing was the main reason mentioned by the participants for unwilling to adopt a child. However, in many cases talking about child adoption are not desirable for them.
